# A phase 2 study of panitumumab with irinotecan as salvage therapy in chemorefractory *KRAS* exon 2 wild-type metastatic colorectal cancer patients

**DOI:** 10.1038/s41416-019-0537-z

**Published:** 2019-07-31

**Authors:** Elena Elez, Carles Pericay, Manuel Valladares-Ayerbes, Inmaculada Bando, Maria Jose Safont, Javier Gallego, Cristina Grávalos, Antonio Arrivi, Alfredo Carrato, Verónica Conde, Maria José Ortiz, Carlos López, Beatriz Alonso, Inmaculada Ruiz de Mena, Eduardo Díaz-Rubio, Josep Tabernero, Enrique Aranda

**Affiliations:** 1 Department of Medical Oncology, Vall d’Hebron Institute of Oncology, Hospital Universitari Vall d’Hebron, Universitat Autònoma de Barcelona, Barcelona, Spain; 2 Department of Medical Oncology, C.S. Parc Tauli, Barcelona, Spain; 3 Department of Medical Oncology, Complejo Hospitalario Universitario A Coruña, A Coruña, Spain; 4 Laboratory Hospital Clínico San Carlos. Instituto de Investigación Hospital Clínico San Carlos (IdISSC), University Complutense, Madrid, Spain; 5 CIBERONC, Madrid, Spain; 6 Department of Medical Oncology, General Universitario de Valencia Hospital, Valencia, Spain; 7 Department of Medical Oncology, General Universitario de Elche Hospital, Alicante, Spain; 8 Department of Medical Oncology, Universitario Doce de Octubre Hospital, Madrid, Spain; 9 Department of Medical Oncology, Son Llàtzer Hospital, Palma de Mallorca, Spain; 10 Department of Medical Oncology, IRYCIS, CIBERONC, Alcalá University, Hospital Universitario Ramón y Cajal, Madrid, Spain; 11 Department of Medical Oncology, Virgen de las Nieves Hospital, Granada, Spain; 12 IMIBIC, Reina Sofía Hospital, University of Córdoba, CIBERONC, Instituto de Salud Carlos III, Córdoba, Spain; 13 Department of Medical Oncology, Marqués de Valdecilla Hospital, Santander, Spain; 14 Department of Medical Oncology, Universitario de Canarias Hospital, Tenerife, Spain; 15 Spanish Cooperative Group for the Treatment of Digestive Tumours (TTD), Madrid, Spain; 16 Present Address: IMIBIC, Reina Sofía Hospital, University of Córdoba, CIBERONC, Instituto de Salud Carlos III, Córdoba, Spain; 17 Present Address: Clínica Rotger, Palma, Balearic Islands, Spain

**Keywords:** Colorectal cancer, Oncology

## Abstract

**Background:**

Targeted agents are standard treatment for *RAS* wild-type metastatic colorectal cancer in the first- and second-line settings. This phase 2 study determined the benefit of targeting the epidermal growth factor receptor (EGFR) with panitumumab plus irinotecan in irinotecan-refractory patients.

**Methods:**

*KRAS* exon-2 wild-type patients failing prior irinotecan received panitumumab (6 mg/kg) and irinotecan (180 mg/m²) every 2 weeks. The primary endpoint was the overall response rate (ORR). Secondary endpoints included safety, progression-free survival (PFS) and overall survival (OS). *KRAS* exon-2 status was evaluated centrally, along with *NRAS*, *BRAF* mutations, *epiregulin*, *amphiregulin*, *PTEN* and *EGFR* copy number status, and correlated with efficacy.

**Results:**

Sixty-one patients were treated. Among the 46 wild-type *RAS* patients, the ORR was 15.2% (seven partial responses), with median PFS of 3.8 months (95% CI 2.7–4.3) and median OS of 12.5 months (95% CI 6.7–15.9). Wild-type *BRAF* patients showed a 13.0% response rate. No significant correlations between response and baseline biomarker expression were identified. Common grade 3–4 adverse events were diarrhoea and rash (18.0% each), hypomagnesaemia and asthenia (8.2% each).

**Conclusions:**

The addition of panitumumab to irinotecan as salvage therapy is feasible but has limited activity in irinotecan-refractory metastatic colorectal cancer. No biomarkers predictive of response were identified.

## Background

Therapeutic management of colorectal cancer (CRC) has changed dramatically over the last few decades with the addition firstly of oxaliplatin and irinotecan to the chemotherapeutic mainstay of fluoropyrimidine with leucovorin, and then subsequently with the use of targeted biological therapies including anti-epidermal growth factor receptor (EGFR) and anti-angiogenic agents, both of which have considerably improved survival outcomes. By consequence, the metastatic chemorefractory setting currently accounts for ~50% of all CRC patients,^1^ and better salvage therapy options are needed for this population.

Among EGFR-targeted therapies, the monoclonal antibodies cetuximab and panitumumab both block EGF and TGFα signalling. Cetuximab was the first to show benefit when added to single-agent irinotecan after fluoropyrimidine-based therapy.^2^ Subsequently, two large phase 3 studies demonstrated that the addition of panitumumab to irinotecan as monotherapy or FOLFIRI in wild-type Kirsten rat sarcoma viral (*KRAS*) patients failing fluoropyrimidine-based therapy improved progression-free survival (PFS) and response rate, although without a significant impact on overall survival (OS).^3,4^ The success of blocking EGFR signalling is dependent on *KRAS* mutational status, with the efficacy benefits of cetuximab treatment in metastatic CRC (mCRC) patients being confined to tumours wild-type for *KRAS* codons 12 and 13, while *RAS* mutations predict adverse outcomes with panitumumab-FOLFOX treatment.^5,6^ Furthermore, benefit with anti-EGFR antibodies in combination with chemotherapy as front-line therapy in patients with *RAS* wild-type mCRC, is greatest in patients with left-sided tumours,^7^ with similar effects in later lines.^8,9^

Few options exist for patients with irinotecan-refractory mCRC. Over a decade ago, the pivotal BOND study demonstrated that the addition of EGFR-targeted cetuximab to irinotecan restored chemotherapy sensitivity in a patient population previously treated with irinotecan, most of whom had received at least two prior therapy lines.^10^ A significantly higher response rate was seen for the combination (22.9% versus 10.8% with irinotecan alone, *p* = 0.007), along with improved PFS (4.1 versus 1.5 months, respectively; hazard ratio 0.54 [95% CI, 0.42–0.71], *p* < 0.001).

In the current study, we report the results of a single-arm phase 2 study evaluating the effect on efficacy of the addition of panitumumab to irinotecan as salvage therapy in wild-type *KRAS* exon-2 mCRC patients progressing on irinotecan-based therapy. Efficacy was analysed in terms of response rate, PFS and OS, along with evaluation of patient characteristics and genetic alterations as potential biomarkers predictive of benefit.

## Methods

### Patients

Adult patients aged ≥18 years with histologically-confirmed metastatic adenocarcinoma of the colon or rectum and wild-type *KRAS* (codons 12 and 13; allelic discrimination, investigator-evaluated) were eligible. Patients had to have progressed (by radiographic imaging) during or within 3 months after irinotecan-based therapy, either 180 mg/m² every 2 weeks (single-agent or FOLFIRI) or 350 mg/m² every 3 weeks (single-agent), and have received irinotecan for at least 6 weeks, with no more than two dose reductions. In addition, one or more measurable lesion, a Karnofsky performance status of at least 70%, adequate haematological, hepatic and renal function, and serum magnesium and calcium levels within normal limits were required. Prior anti-EGFR therapy was not permitted. Patients provided written informed consent prior to enrolment.

### Study design

This phase 2 single-arm, open-label study was performed in 12 Spanish centres. Patients received panitumumab (6 mg/kg, 60-min infusion) followed by irinotecan (180 mg/m², 90-min infusion) every 2 weeks. For patients who had received a reduced dose with prior irinotecan therapy, this dose was maintained, and for patients who had received 350 mg/m² irinotecan every 3 weeks, the equivalent every-2-weeks dose was used. In the event of grade 3–4 related events or skin or nail toxicity requiring treatment or considered intolerable, panitumumab was withheld and the dose reduced (to 4.8 then 3.2 mg/kg), while irinotecan was maintained. If irinotecan was delayed, panitumumab was also delayed (maximum of 1 month). Panitumumab monotherapy was permitted after irinotecan discontinuation but not vice versa. Patients who underwent curative metastatic resection could continue in the study 4 weeks later. Patients continued treatment until progression or unacceptable toxicity.

### Efficacy and safety assessments

Tumour response was evaluated by computerised tomography scan and/or magnetic resonance imaging according to the modified Response Evaluation Criteria in Solid Tumours (m-RECIST).^11^ Response was assessed every 6 weeks during the first 6 months and every 2 months thereafter until progression or withdrawal. Responses were confirmed at least 1 month after the criteria were first met. After discontinuation, patients without progression were followed-up every 6 weeks until progression, and progressing patients were followed-up every 3 months. Adverse events (AEs) were graded according to NCI-CTCAE v3.0.

### Biomarker analysis

Tumour blocks were reviewed centrally. DNA and RNA were extracted using QIAamp^®^ DNA FFPE Tissue and RNeasy^®^ FFPE kits and analysed with a Nanodrop^®^ ND1000. Mutations in *KRAS* codons 61, 117 and 146, and *NRAS* codons 12, 13, 61, 117 and 146 were detected by pyrosequencing. Mutations in *BRAF* (V600E) and *PIK3CA* (R88Q, N345k, C420R, E542K, E545D, E545K, M1043I, H1047R and H1047Y) were detected by real-time PCR cobas^®^ Mutation Tests. *Amphiregulin* and *epiregulin* mRNA expression was evaluated by real-time PCR with TaqMan^®^ Gene Expression assays. ROC curves were used to determine cut-off values for high versus low expression. PTEN protein expression was assessed with the 17.A mouse monoclonal antibody. PTEN-negative was defined as no or weak staining and positive as moderate or strong. *EGFR* was analysed by fluorescence in situ hybridisation by two blinded pathologists using an *EGFR*-specific probe (orange signal) and a control chromosome probe 7 (green signal); two orange and green signals per tumour cell or a ratio ≤1 was considered to be no *EGFR* amplification, more than two orange and green signals with a ratio greater than 2 or a ratio of 1.5 in ≥10% cells was considered amplification, four orange and green signals in ≥10% tumour cells was polysomy. See also Supplementary Methods.

### Statistical analysis

A two-stage Simon model was used to test the null hypothesis that P_0_ ≤ 0.15^12^ versus true activity with P_1_ ≥30%, and assuming α = 0.1 and β = 0.1. Accordingly, if responses were seen in at least six of the first 34 evaluable patients, a further 19 evaluable patients were included. If overall, at least 16 patients achieved an objective response, the combination was considered sufficiently active. Sixty wild-type *KRAS* patients were planned, allowing for a 10% non-evaluable rate.

The primary endpoint was the overall response rate (ORR) in the intention-to-treat (ITT) population. Secondary endpoints included disease control rate, PFS, OS, and safety. PFS and OS were analysed by the Kaplan–Meier method. Efficacy was also analysed in terms of candidate predictive molecular markers (*RAS*, *BRAF*, *PI3K* and *PTEN* mutations, *EGFR* amplification, *PTEN* loss-of-function, and *epiregulin* and *amphiregulin* levels) using log-rank tests, and baseline patient and disease covariates using logistic regression with univariate and multivariate proportional hazard models. Analyses were performed with SAS version 9.4.

## Results

A total of 61 patients with *KRAS* exon 2 wild-type CRC tumours were enrolled between July 2009 and June 2012 and received a median of 78 days of treatment (range, 1–279). Patient demographics and disease characteristics are shown in Table 1. All patients had received one or two prior lines of treatment for metastatic disease, all of whom had received prior irinotecan, 92% of patients had received prior oxaliplatin and 62% had been treated with at least one prior line of bevacizumab. Extended *RAS* analysis was performed in 57 patients, 46 of whom were confirmed to be wild-type.

**Table 1:** Patient, tumour and treatment characteristics, ITT population

	*N* = 61
Age, in years	
Median (range)	65 (40–81)
>70 years, *N* (%)	20 (32.8%)
Males, *N* (%)	40 (65.6%)
Primary tumour, *N* (%)	
Colon	43 (70.5%)
Rectum	18 (29.5%)
Median time since diagnosis in months, (range)	30 (3–130)
TNM stage, *N* (%)^a^	
II	5 (8.2%)
III	9 (14.8%)
IV_A-B_	43 (7.5%)
Histology grade, *N* (%)^b^	
1	11 (18.0%)
2	35 (57.4%)
3	9 (14.8%)
Wild-type *RAS*, *N* (%)^c^	46 (80.7%)
Metastatic locations, *N* (%)	
Liver	44 (72.1%)
Lung	35 (57.4%)
Lymph nodes	18 (29.5%)
Peritoneum	8 (13.1%)
Other	17 (27.9%)
Prior palliative therapy, *N* (%)	
N lines	
1	61 (100%)
2	43 (70.5%)
≥3	11 (18.0%)
Oxaliplatin	56 (92.0%)
Bevacizumab	38 (6.02%)
Prior adjuvant therapy, *N* (%)	24 (39.3%)
Prior surgery, *N (%)*	50 (82.0%)

^a^ Missing data for four patients

^b ^Missing data for six patients

^c ^Extended *RAS* analysis was performed in 57 patients

### Anti-tumour activity

At the cut-off date of 27 February 2014, median follow-up was 11 months (95% CI 6.7–14.8). In the overall population, eight patients had partial responses giving an ORR of 13.1% (95% CI, 4.6– 21.6%) in the 61 ITT patients (Table 2). The disease control rate was 62.3% (95% CI, 50.1–74.5%), including 30 patients with stable disease, lasting a median of 3.0 months (95% CI, 2.1–3.4 months). Median PFS was 3.7 months (95% CI, 2.7–4.2 months) and median OS was 11.1 months (95% CI, 7.1–14.8 months). None of the candidate predictive factors evaluated by regression analysis (sex, performance status, age, number of previous lines of therapy, tumour location, lactate dehydrogenase levels) affected survival parameters or the response rate.

**Table 2:** Tumour response (m-RECIST) and survival, ITT patients

	All patients *N* = 61	Wild-type RAS *N* = 46	Wild-type RAS with prior bevacizumab *N* = 29
Objective response, *N* (%)			
Partial response	8 (13.1%)	7 (15.2%)	6 (20.7%)
Stable disease	30 (49.2%)	24 (52.2%)	14 (48.3%)
Progressive disease	17 (27.9%)	12 (26.1%)	8 (27.6%)
Not evaluable/not done	6 (9.8%)	3 (6.5%)	1 (3.5%)
Overall response rate [95% CI]	13.1% [4.6–21.6%]	15.2% [4.8–25.6%]	20.7% [6.0–35.4%]
Disease control, *N* (%) [95% CI]	38 (62.3%) [50.1–74.5%]	31 (67.4%) [53.8–80.4%]	20 (69.0%) [52.1–85.8%]
PFS (months)			
Median [95% CI]	3.7 [2.7–4.2]	3.8 [2.7–4.3]	3.8 [2.4–4.6]
N events/censored	60/1	45/1	29/0
OS (months)			
Median [95% CI]	11.1 [7.1–14.8]	12.5 [6.7–15.9]	14.8 [6.7–16.7]
N events/censored	56/5	42/4	28/1

**Fig. 1:**
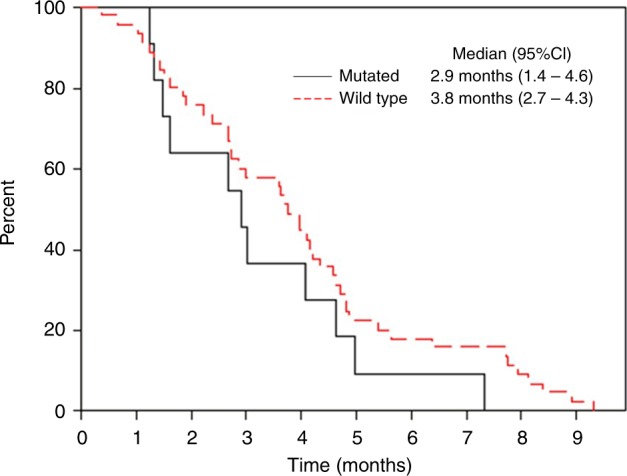
Progression-free survival in *RAS* wild-type and mutated ITT patients, by Kaplan–Meier estimate

Efficacy was also analysed among the 46 *RAS* wild-type patients, seven of whom had partial response versus 1 of the 11 *RAS*-mutated patients, giving ORRs of 15.2% (Table 2) versus 9.1%, in these two populations, respectively. Disease control in the wild-type population was 67.4% (95% CI, 53.8–80.4%). This translated into only small differences in survival, with a median PFS in wild-type patients of 3.8 months (95% CI, 2.7–4.3 months) versus 2.9 months (95% CI, 1.4–4.6 months) in *RAS*-mutated patients (Fig. 1). Median OS was 12.5 months (95% CI, 6.7–15.9 months) in wild-type patients versus 11.1 months (95% CI, 4.2–23.9 months) in mutated patients. Efficacy was further analysed in this sub-population according to prior bevacizumab treatment, showing similar trends to the overall wild-type population (Table 2). Although ORR was improved in patients who had received prior bevacizumab compared to those who had not (20.7% vs 5.9%, respectively), this did not translate into any obvious differences in terms of disease control (69.0% vs 64.7%, respectively) or median PFS (3.8 vs 3.9 months, respectively).

Efficacy was also analysed according to several candidate predictive biomarkers, comparing subpopulations with and without mutations or amplifications in key molecular pathways (*BRAF*, *PI3KCA*, PTEN and *EGFR*) and with high versus low expression (*amphiregulin* and *epiregulin*). No significant associations between the presence of these biomarkers and response were found, although analyses suggested some minor trends towards improved efficacy in patients with wild-type *BRAF*, wild-type *PI3KCA*, high *epiregulin* or *amphiregulin* expression and/or without *EGFR* amplification.

Among the 54 patients with wild-type *BRAF* status, seven had a partial response, giving a response rate of 13.0% versus 25.0% in mutated patients (one response among four patients). PFS was 3.7 months (95% CI, 2.7–4.2) versus 1.8 months (95% CI, 0.7–6.4), respectively. Analysis of the all-*RAS*/all-*BRAF* wild-type population gave similar outcomes to each of the individual contributing wild-type populations with an ORR of 14.3%, disease control of 71.4% (95% CI 57.8–85.1%) and median PFS of 4.0 months (95% CI, 2.7–4.6 months). Of the 49 wild-type *PI3KCA* patients, seven had partial response, giving a response rate of 14.3% versus 12.5% in mutated tumours. PFS was 3.9 months (95% CI, 2.9–4.3) versus 2.7 months (95% CI, 0.7–5.7) respectively.

*Epiregulin* was analysed in 35 patients; partial responses were seen in three of the 18 high expression patients and two of the 17 low expression patients, giving 16.7% and 11.8% response rates, respectively. Median PFS was 3.9 months (95% CI, 2.7–4.6) and 2.9 months (95% CI, 1.4–4.8), respectively. Among the 35 patients evaluated for amphiregulin, four partial responses were seen in the 18 high expression patients and 1 of the 17 patients with low expression, giving response rates of 22.2% and 5.9%, respectively. Median PFS was 4.3 months (95% CI, 3.0–5.4) and 2.7 months (95% CI, 1.4–3.6), respectively.

Among the 32 patients evaluable for PTEN, none of the nine patients expressing PTEN responded, while five non-expressing patients had a partial response (21.7%). Median PFS was 4.1 months (95% CI, 0.7–4.9) in patients with PTEN and 3.0 months (95% CI, 1.6–4.1) in patients without. Among the 34 patients evaluable for *EGFR* amplification status, three patients showed amplification, all of whom had presented stable disease, compared with five partial responses among the 31 *EGFR*-negative patients (16.1%). PFS was 2.9 months (95% CI, 2.7–3.6) versus 3.7 months (95% CI, 1.6–4.6).

### Safety

As anticipated, the most common treatment-related AEs were gastrointestinal and dermatological, including diarrhoea (62.3%), rash (59.0%), asthenia (50.8%), hypomagnesaemia (44.3%), mucosal inflammation (29.5%), vomiting (26.2%) and nausea (24.6%) (Table 3). Dry skin, paronychia and acne were reported in 26.2%, 21.3% and 18.0% of patients, respectively. The profile of grade 3–4 related AEs was similar with lower frequency, including diarrhoea and rash (18.0%), and hypomagnesaemia and asthenia (8.2% each). One patient died as a result of sepsis considered related to irinotecan, with concurrent pneumonia and grade 4 febrile neutropenia. Eleven patients (18.0%) had a related AE leading to treatment discontinuation, five patients stopped panitumumab and six patients stopped irinotecan due to related toxicity.

**Table 3:** Main treatment-related adverse events (NCI-CTCAE) (*N* = 61)

	Grade 1–4	Grade 3	Grade 4
Diarrhoea	38 (62.3%)	9 (14.8%)	2 (3.3%)
Rash	36 (59.0%)	11 (18.0%)	
Asthenia	31 (50.8%)	5 (8.2%)	
Hypomagnesaemia	27 (44.3%)	3 (4.9%)	2 (3.3%)
Mucosal inflammation	18 (29.5%)		
Dry skin	16 (26.2%)	2 (3.3%)	
Vomiting	16 (26.2%)		
Nausea	15 (24.6%)		
Paronychia	13 (21.3%)	1 (1.6%)	
Alopecia	12 (19.7%)	1 (1.6%)	
Acne	11 (18.0%)		
Neutropenia	9 (14.8%)	2 (3.3%)	2 (3.3%)
Decreased appetite	7 (11.5%)	1 (1.6%)	
Anaemia	6 (9.8%)	1 (1.6%)	
Erythema	6 (9.8%)		
Abdominal pain	5 (8.2%)	1 (1.6%)	
Conjunctivitis	5 (8.2%)	1 (1.6%)	
Hypokalaemia	4 (6.6%)	1 (1.6%)	
Gastrointestinal toxicity	3 (4.9%)	1 (1.6%)	
Back pain	2 (3.3%)	1 (1.6%)	
Keratitis	1 (1.6%)		1 (1.6%)
Acute respiratory distress	1 (1.6%)	1 (1.6%)	
Bacterial infection	1 (1.6%)	1 (1.6%)	
Dermatitis acneiform	1 (1.6%)	1 (1.6%)	
Intestinal obstruction	1 (1.6%)	1 (1.6%)	
Infusion reaction	1 (1.6%)	1 (1.6%)	
Toxic skin eruption	1 (1.6%)	1 (1.6%)	

## Discussion

In this single-arm combination study, the addition of panitumumab to irinotecan in irinotecan-refractory wild-type *KRAS* exon-2 mCRC patients gave a 15% response rate. This falls well short of the protocol’s statistical hypothesis threshold of 30% (reflecting 16 responses out of 53 patients), which was considered to demonstrate an efficacy benefit. It is also notably lower than the 35% response rate reported in heavily pre-treated patients harbouring wild-type *KRAS* (codon 12 and 13) treated with panitumumab and irinotecan in the one-arm phase 2 French GERCOR study, as was PFS (6.3 months in the GERCOR versus 3.8 months in our study).^13^ This response rate was also slightly lower than that reported in an equivalent approach and setting in a phase 2 Japanese study, TOPIC, in which the response rate was 23%,^14^ and was also lower than the 26% rate reported in a preliminary evaluation of another phase 2 randomised Japanese study, WJOG6510G, comparing this EGFR combination with the equivalent cetuximab combination.^15^ Median PFS and OS in our study (3.8 and 12.5 months, respectively) were also shorter than those reported in the WJOG6510G study (5.4 and 14.9 months, respectively), but in contrast were improved compared to the TOPIC study (2.7 and 7.3 months, respectively). Differences in irinotecan regimens may have influenced response rates in each of these studies, as suggested from analyses performed in the TOPIC study.

The 15% response rate with the combination therapy is also lower than the 22% rate reported for single-agent panitumumab in the phase 3 ASPECCT study in a comparison with single-agent cetuximab in chemotherapy-refractory wild-type *KRAS* exon 2 mCRC patients,^16^ although more patients in our study had received prior bevacizumab. Median PFS and OS in our study were similar to those reported in the ASPECCT study (4.1 and 10.4 months, respectively), as well as to those reported in *KRAS* wild-type patients treated with panitumumab and best supportive care in a phase 3 study (response rate 27%, median PFS 3.8 months, median OS 10.0 months).^17^ In light of all these studies, our results suggest that the addition of panitumumab to irinotecan as salvage therapy did not offer a meaningful efficacy advantage in the setting of our study.

It is important to bear in mind that since 2015, recommendations for treatment of mCRC patients have included that EGFR inhibitors should not be administered in cases of *NRAS*-mutated tumours.^18^ However as this had not yet been formally implemented at the time of patient accrual during the current study (2009–2012), *NRAS*-mutated patients were included and treated. The absence of biomarkers for response other than *KRAS*, is a pressing issue that needs to be resolved in order to move ahead with the approach of optimising the choice of therapy, and to ensure avoiding unnecessary drug exposure and to overcome resistance developing after treatment with targeted therapies. The recent phase 2 randomised AGITG ICECREAM trial evaluated the addition of irinotecan to cetuximab in chemorefractory mCRC patients, and demonstrated a 36% response rate in patients who were quadruple wild-type for *KRAS*, *NRAS*, *BRAF* and *PI3KCA* after treatment with irinotecan and cetuximab, and a 6-month PFS rate of 41%.^19^ Accumulating evidence suggests that response to panitumumab in advanced CRC correlates with wild-type *BRAF*,^20^ *EFGR* copy number,^21^ *epiregulin* and *amphiregulin* levels,^22^ while *BRAF*, *NRAS* and *PIK3CA* mutations and non-functional PTEN have all been associated with resistance to anti-EGFR therapies.^23^ Although minor non-significant trends in this study suggested a greater benefit in patients with wild-type *BRAF*, wild-type *PI3KCA*, high *epiregulin* or *amphiregulin* expression and/or without *EGFR* amplification, none of the members of the RAS/RAF/ERK and PI3K/PTEN pathways were definitively identified as potential predictive biomarkers. Nonetheless the French GERCOR study argues for the value of salvage panitumumab with irinotecan in patients wild-type for multiple *EGFR* markers, in light of the 46% response rate and the median PFS of 8.7 months seen in patients without mutations in any of the rare *KRAS*, *NRAS* and *BRAF* genes, while no responses were seen in patients with confirmed mutations.^13^

At the time this study was launched, none of the salvage therapies used were considered standard of care in mCRC patients who had failed the classic chemotherapeutic treatments of fluoropyrimidine, irinotecan and oxaliplatin in combination with targeted therapies. In the most recent consensus guidelines for managing mCRC,^1,24^ cetuximab combined with irinotecan is recommended in irinotecan-refractory patients. The multi-targeted kinase inhibitor regorafenib has recently been approved for salvage therapy given the significant survival advantage seen over best supportive care.^25^ Similarly, the trifluridine/tipiracil combination is also recommended in the salvage setting, following results suggesting a similar benefit with less toxicity.^26^

The toxicity profile of the combination was as expected, with effects similar to those reported previously for panitumumab addition to irinotecan^3,4,13,14^ as well as that reported in the BOND study when cetuximab was co-administered with irinotecan in this setting.^10^ Dermatological toxicity frequently associated with EGFR blockade was prevalent, but was generally well managed with dose modifications.

It should be acknowledged that the use of a single-arm design and the relatively small sample size (and small number of responses) limit interpretation of the current study, notably in terms of identification of predictive and pharmacodynamic biomarkers. Future studies in this setting should exploit the addition of other targeted therapies to panitumumab. The use of circulating cell-free DNA in liquid biopsies is now widely considered a reliable method for determining *KRAS* and *BRAF* gene mutations in mCRC.^27^ Use of liquid biopsies is becoming increasingly widespread, to facilitate routine and prospective biomarker testing in clinical studies, and to overcome the difficulties associated with obtaining biopsies of adequate quality or serial biopsies.

In conclusion, the addition of panitumumab to irinotecan was feasible as salvage therapy in a heavily pre-treated population of mCRC, in which most patients had received at least two prior lines of therapy including the anti-angiogenic agent bevacizumab. Nonetheless, the value of the panitumumab/irinotecan combination over single-agent panitumumab appears limited.

## Supplementary information

Materials and Methods – Biomarkers analyses

## Data Availability

Data and results are available at the Department of Medical Oncology, Vall d’Hebron Institute of Oncology, Universitat Autònoma de Barcelona, Barcelona, Spain.
