# Supplementary material for: A phase 2 study of panitumumab with irinotecan as salvage therapy in chemorefractory KRAS exon 2 wild-type metastatic colorectal cancer patients
Source: Br J Cancer. 2019 Jul 31;121(5):378–83. doi: 10.1038/s41416-019-0537-z (PMC6738054; doi:10.1038/s41416-019-0537-z)
Supplement: Supplementary file 1 — Materials and Methods – Biomarkers analyses [file 41416_2019_537_MOESM1_ESM.doc]

***Supplementary Data***

**Materials and Methods – Biomarkers analyses**

Formalin-fixed, paraffin-embedded tumour blocks were reviewed centrally. DNA and RNA were extracted using QIAamp® DNA FFPE Tissue and RNeasy® FFPE Kits (Qiagen, Madrid, Spain) and analysed with a Nanodrop® ND1000 (Thermo Fisher Scientific, Fremont, CA). Mutations in *KRAS* exons 3 (codon 61) and 4 (codons 117 and 146), *NRAS* exons 2 (codons 12 and 13), 3 (codon 61) and 4 (at codons 117 and 146) were detected by Pyrosequencing using RAS Extension Pyro and NRAS Pyro Kits (Qiagen, Hilden, Germany). Mutations in *BRAF* exon 15 (V600E) and *PIK3CA* exons 1 (R88Q), 4 (N345k), 7 (C420R), 9 (E542K, E545D, E545K) and 20 (M1043I, H1047R, and H1047Y) were detected by real-time PCR cobas® Mutation Tests on a cobas® Z480 Analyser (Roche Molecular Systems, Inc., Branchburg, NJ, USA). *Amphiregulin* and *epiregulin* mRNA expression was evaluated by RT-PCR. cDNA was prepared with the Superscript III Reverse Transcriptase (Invitrogen, Parsley, UK) as described previously [1]. Quantification (including the *PSMB4* internal reference) was done with TaqMan Gene Expression assays using the ABI PRISM 7500 Fast Real Time PCR System (Life Technologies, Foster City, CA, USA). Assay IDs were Hs00154995_m1 for *epiregulin*, Hs00155832_m1 for *amphiregulin* and Hs00160598 for PSMB4. Relative gene expression quantification was performed in triplicate according to the comparative Ct method using *PSMB4* as the endogenous control and a pool of cDNA from 11 colon tissue samples of healthy individuals as an internal control. The 2^-ΔΔCT^ number was used for relative mRNA quantification, where ΔCT is the difference between the Ct values of the target and endogenous genes. ΔΔCT is the difference between ΔCT for each sample and ΔCT of the internal control. ROC curves were used to determine the cut-off values for high versus low expression.

PTEN protein expression was assessed in tissue sections using the 17.A mouse monoclonal antibody (1:25 dilution, Neomarkers; ThermoFisher Scientific Inc., Fremont, CA), as previously described [2]. After deparaffinization and hydration of sections, antigens were unmasked by heat in EDTA buffer. Immunostaining was performed using the UltraVision LP Large Volume Detection System AP Polymer (Thermo Scientific, Waltham, MA, USA). Negative controls were prepared by omitting the primary antibody. Prostate cancer was used as external positive controls and endothelial cells were used as internal positive controls. As described previously [3], intensity was scored according to a four-tier system: 0, no staining; 1, weak; 2, moderate; and 3, strong. PTEN-negative was defined as no staining (0) plus weak staining (1) and positive as moderate staining (2) plus strong staining (3).

*EGFR* was analysis by fluorescent *in situ* hybridization (FISH) on tissue sections using an *EGFR*-specific sequence probe (LSI EGFR SpectrumOrange) and control chromosome probe 7 (CEP7- SpectrumGreen), according to the manufacturers recommended protocol (Vysis-Abbott Molecular Diagnostics, USA). The CEP 7 probe showing green signal indicates the chromosome 7 centromere, and the *EGFR* probe shows orange signal representing the *EGFR* gene copy number. Two blinded pathologists evaluated alterations in EGFR gene copy number. The presence of only two orange and green signals in each tumour cell or a ratio ≤1 was considered to be no *EGFR* amplification; the presence of four signals in ≥ 10% tumour cells was considered as polysemy; the presence of more than two orange and green signals in each tumour cell with a ratio greater than 2 or 1.5 in ≥ 10% cells was considered gene amplification.

**Supplementary Data References**

1. Romero A, Martín M, Cheang MCU, et al: Assessment of Topoisomerase II α Status in Breast Cancer by Quantitative PCR, Gene Expression Microarrays, Immunohistochemistry, and Fluorescence in Situ Hybridization. Am J Pathol 178:1453–1460, 2011

2. Frattini M, Signoroni S, Pilotti S, et al: Phosphatase Protein Homologue to Tensin Expression and Phosphatidylinositol-3 Phosphate Kinase Mutations in Colorectal Cancer. Cancer Res 65:11227–11227, 2005

3. Loupakis F, Pollina L, Stasi I, et al: PTEN expression and KRAS mutations on primary tumors and metastases in the prediction of benefit from cetuximab plus irinotecan for patients with metastatic colorectal cancer. J Clin Oncol 27:2622–2629, 2009
